# Weighted gene co-expression network analysis revealed T cell differentiation associated with the age-related phenotypes in COVID-19 patients

**DOI:** 10.1186/s12920-023-01490-2

**Published:** 2023-03-25

**Authors:** Yao Lin, Yueqi Li, Hubin Chen, Jun Meng, Jingyi Li, Jiemei Chu, Ruili Zheng, Hailong Wang, Peijiang Pan, Jinming Su, Junjun Jiang, Li Ye, Hao Liang, Sanqi An

**Affiliations:** 1 Medical Laboratory Centre, Life Sciences Institute, Guangxi Medical University, Nanning, 530021 Guangxi China; 2 Department of Biochemistry and Molecular Biology, School of Basic Medicine, Guangxi Medical University, Nanning, 530021 Guangxi China; 3 Biosafety Level 3 Laboratory and Guangxi Collaborative Innovation Centre for Biomedicine, Guangxi Medical University, Nanning, 530021 Guangxi China

**Keywords:** COVID-19, Weighted gene, Aging, T-cell immunity

## Abstract

The risk of severe condition caused by Corona Virus Disease 2019 (COVID-19) increases with age. However, the underlying mechanisms have not been clearly understood. The dataset GSE157103 was used to perform weighted gene co-expression network analysis on 100 COVID-19 patients in our analysis. Through weighted gene co-expression network analysis, we identified a key module which was significantly related with age. This age-related module could predict Intensive Care Unit status and mechanical-ventilation usage, and enriched with positive regulation of T cell receptor signaling pathway biological progress. Moreover, 10 hub genes were identified as crucial gene of the age-related module. Protein–protein interaction network and transcription factors-gene interactions were established. Lastly, independent data sets and RT-qPCR were used to validate the key module and hub genes. Our conclusion revealed that key genes were associated with the age-related phenotypes in COVID-19 patients, and it would be beneficial for clinical doctors to develop reasonable therapeutic strategies in elderly COVID-19 patients.

**Supplementary Information:**

The online version contains supplementary material available at 10.1186/s12920-023-01490-2.

## Background

The Corona Virus Disease 2019 (COVID-19) pandemic caused by the severe acute respiratory syndrome coronavirus 2 (SARS-CoV-2) has had a significant impact on society and the economy worldwide, resulting in a large number of deaths. It has been identified that age-related vulnerability is a key factor in the burden of disease during this pandemic [1]. Research has shown that COVID-19 mainly affects the elderly population and many risk factors, such as advanced age [2], male gender, and clinical complications, contribute to the progression of the disease to severe and critical stages [3]. Despite the efforts made in researching and developing vaccines and drugs, COVID-19 has not been well controlled or cured, and there are still many irreversible complications [4–6]. Therefore, it is crucial to investigate the spectrum and pathogenesis of the disease in susceptible populations during this pandemic.

Macrophage infiltration into the lung cause a rapid and intense cytokine storm in COVID-19 patients, ultimately leading to complications such as metabolic syndrome, obesity, type 2 diabetes, lung disease, and cardiovascular disease. These age-related diseases increase the severity and lethality of COVID-19, and in some cases, can even lead to multi-organ failure and death [7]. It is widely accepted that elderly patients with COVID-19 are more likely to progress to a severe condition and unfavourable outcome due to age-related biological alterations [2, 8, 9]. The underlying mechanisms that cause different outcomes in different age groups may include age-related pattern recognition receptor activation, type 1 interferon activation, functionally impaired neutrophils, macrophages, T cells response, and other complex progressive changes in the immune system [10, 11]. However, the exact mechanism is still unclear, and it is urgent for us to investigate the most important factor causing different outcomes in different age groups of COVID-19 patients.

Several computational and research methods have been developed in order to understand the pathogenesis of specific disease states. Part of these methods are used to explore underlying gene networks, which are useful in guiding diseases understanding and their mechanistic pathways. Notably, co-expression analysis is one of many approaches. In this analysis, clusters genes into co-expression groups are called modules. Genes which belong to the same module are considered to have the same functional properties [12]. By using the concepts of graph-theory, it enables researchers to systematically understand the relationship between module genes and phenotypes based on module characteristic genes [12]. Indeed, weighted correlation network analysis (WGCNA) of co-expression analysis has been applied to many biological processes [13–18]. In short, gene networks provide tools to perform group level gene comparisons and identify biologically relationships between genes and phenotypes comprehensively.

Therefore, in this study, we analysed age-related gene networks through co-expression analysis in 100 COVID-19 patients. The SRP279280 dataset was used to identify the gene module which tightly connected with age trait. The function and hub genes of the key module were identified, the protein–protein interaction network and transcription factor-gene interaction were constructed. The results of our study may provide new insights into the mechanisms underlying adverse progression of COVID-19 in advanced age and may help identify potential diagnostic biomarkers and therapeutic targets.

## Methods

### Data collection and pre-processing

The gene expression profile and clinical information of GSE157103 were downloaded from publicly Sequence Read Archive (SRA) database SRP279280 (https://www.ncbi.nlm.nih.gov/sra/). Dataset GSE157103 contained a total of 100 COVID-19 patients’ normalized peripheral blood mononuclear cell (PBMC) data in vivo, including 62 males and 38 females, 50 ICU-patients and 50 non-ICU-patients, 42 mechanical-ventilation-patients and 58 non-mechanical-ventilation-patients [19]. Of note, there was a sample for which age information was missing. The age of rest samples ranged from 21 to 90. The 10,985 genes whose transcript per million (TPM) was more than 1 were selected for further analysis from all 19,472 gene set in expression profile. Average method was used to calculate the distance between samples, and 3 samples were eliminated as outliers.

In addition, independent dataset, which was collected from the research of Huang et al. [20], are used to verify the consistency and reliability of our results. It contains 4 mild COVID-19 patients’ and 4 severe COVID-19 patients’ normalized PBMC RNA-seq data.

### Weighted gene co-expression network analysis and screening for key modules

The WGCNA R package (version 1.7.1) based on R (version 4.2.1) were performed to identify key genes associated with different age groups in dataset GSE157103 COVID-19 patients [19]. WGCNA is the most widely used methods to identify genes and modules associated with specific phenotypes [21]. All genes and samples were checked for excessive deletion values and for abnormal samples. And three outliers were cut out. R^2^ was set as 0.8 to weight highly correlated genes, and the soft thresholding power (β) was 14. For the purpose of defining clusters of genes and getting a more suitable number of modules, the minimal module size was set as 30, mergeCutHeight was set as 0.05 and other parameters retain the default values. The adjacency matrix was used to calculate the topological overlap matrix (TOM) and a heatmap was used to show the degree of overlap in share neighbours between pairs of genes in the network.

In order to identify the key module with significant correlation, we calculated the correlation between each module eigengenes connectivity and the age of samples. The correlation coefficient values were displayed within a heatmap. The module that was most significantly associated with age was considered as the key module. The correlation analysis in this study were performed using the Pearson correlation as described in the “WGCNA” package [12].

### Functional enrichment analysis and immune evaluation of key module

The enrichment analysis was performed to explore the functions of the key modules. DAVID (https://david.ncifcrf.gov/) [22] is a powerful tool for gene functional annotation and analysis. The comprehensive resource such as Kyoto Encyclopedia of Genes and Genomes (KEGG) [23–26], Gene Ontology (GO) etc. datasets were included in the enriched pathways. The Benjamini–Hochberg (BH) adjusted *p*-values were sorted for visualization.

We used single-sample GSEA (ssGSEA) which is the measure of genes that are co-ordinately up- or down-regulated within a sample to calculate immune enrichment scores for each sample. ssGSEA define an enrichment score that represents the absolute degree of enrichment of the gene set in each sample within a dataset. Sequencing normalization of gene expression values was performed, and enrichment scores were generated using the empirical cumulative distribution function (ECDF) of the genes in the signature and the remaining genes. We used the immune cell set as a reference to perform immune scores between 100 samples.

### Principal component analysis and receiver operating characteristic curve analysis

Principal component analysis (PCA) is one of the most widely applied data dimensionality reduction algorithms [27]. It was used to remove noise and unimportant features and retain the most important features of the key module. The key module genes with possible correlation were transformed into linearly uncorrelated variables through orthogonal transformation, and the first principal component (PC1) was chosen for further analysis according to the contribution rate. The first two principal components were selected for two-dimensional visualization. To further evaluate the potential diagnostic value of the first principal component in key module for COVID-19, the receiver operating characteristic (ROC) curve analysis was performed by using the pROC (version 1.18.0) [28] routine in R. We evaluated the diagnostic efficacy of PC1 and age for sex, ICU admission, and mechanical-ventilation.

### Protein–protein interaction analysis and network construction

In biological systems, protein–protein interaction (PPI) networks are evaluated and analysed to understand the interaction between intracellular protein, which can improve the comprehension of protein function. The key module genes were used to construct PPI network through STRING web tools [29], and all parameters were set to default values. STRING provides the PPI network that shows how the identified hub genes (proteins) interrelate functionally and physically with each other through encoding the gene list as input [30]. The PPI results were analysed and visualized by Cytoscape (V3.8.2) [31–33]. The hub genes were chosen based on degree connectivity via cytoHubba [34] in Cytoscape. Among the 11 methods in Cytoscape’s plug-in ctyoHubba, Maximal Clique Centrality (MCC) performs the best, with the advantage of precision predictive function in essential proteins from the Yeast PPI network [34]. After MCC algorithm, all module genes were ranked according to their intramodular connectivity, and only the top ten genes were selected as hub genes.

### Transcription factors-genes interactions

Transcription Factors (TFs) are proteins that bind DNA and regulate transcription in a sequence specified manner. In order to further understand the regulation of hub genes, we found out the TFs of the hub genes and constructed the TFs-genes network map through NetworkAnalyst 3.0 (https://www.networkanalyst.ca/) [35]. The TFs and gene targets data were obtained based on the ENCODE ChIP-seq database (https://www.encodeproject.org/) [36]. By BETA Minus algorithm, only peak intensity signal < 500 and the predicted regulatory potential score < 1 was included.

### Collecting experimental samples, isolating PBMC and extracting total RNA

5 mL of fasting elbow venous blood was collected from 6 samples (3 samples in 20–30 years old considered as younger group and 3 samples in 40–50 years old considered as older group) and centrifuged at 800 g/min at 25 °C for 8 min. The supernatant was thoroughly mixed with the same amount of PBS, 6 mL of Ficoll separation solution was added and centrifuged at 1200 g/min at 25 °C for 10 min. The PBMC was absorbed and placed in a new 50 mL centrifuge tube, thoroughly mixed with PBS by 5 times, centrifuged at 300 g/min at 25 °C for 10 min. The supernatant was discarded and 2 mL RBC cracking solution was added into the tube, stood at room temperature for 5 min. An appropriate amount of PBS was added and centrifuged at 2500 rpm/min at 25 °C for 5 min. The supernatant is discarded and the precipitation is retained. Trizol (American ambion) was added into the precipitation, and the total RNA was extracted by Trizol method. Then the dissolved RNA was transferred to the ice box, and the concentration and quality of total RNA were evaluated by NanoDrop ND-2000 spectrophotometer. The samples were stored in the refrigerator at − 80 °C for further using.

### RT-qPCR

Total RNA was reverse-transcribed into cDNA using PrimerScript RT Master Mix kit (Japan Takara), and then real-time fluorescent quantitative PCR was performed according to the instructions of TB Green Premix Ex Taq II kit (Japan Takara). The primers were synthesized by Shanghai Shengong Biological Engineering Company, and the sequence was:

1. *RORC*: upstream primer 5ʹ-AGCGCTCCAACATCTTCTCC-3ʹ, downstream primer 5ʹ-ACCACTTCCATTGCTCCTGC-3ʹ,
2. *CCR7*: upstream primer 5ʹ-TTCCAGCTGCCCTACAATGG-3ʹ, downstream primer 5ʹ-CAAGAAAGGGTTGACGCAGC-3ʹ;
3. *MYC*: upstream primer 5ʹ-GGCTCCTGGCAAAAGGTCA-3ʹ, downstream primer 5ʹ-CTGCGTAGTTGTGCTGATGT-3ʹ.

The reaction total volume was 20 uL of PCR reaction system, including SYBR Premix Ex Taq 10 uL, upper and downstream primers 0.4uL of each, ROX Reference DyeII 0.4 uL, cDNA template 2 uL and sterilized distilled water supplement system to 20 uL. Using GAPDH as the internal reference, the experiment was repeated for 3 times to take the mean value, and the relative mRNA expression was calculated by 2-ΔΔCt method.

SPSS 23.0 and Graphpad prism 9.0 were used for statistical analysis, and T-test analysis was performed for comparison between groups. *p* < 0.05 was considered statistically significant.

## Results

### Identification of key module with highest correlation with age phenotype

**Fig. 1:**
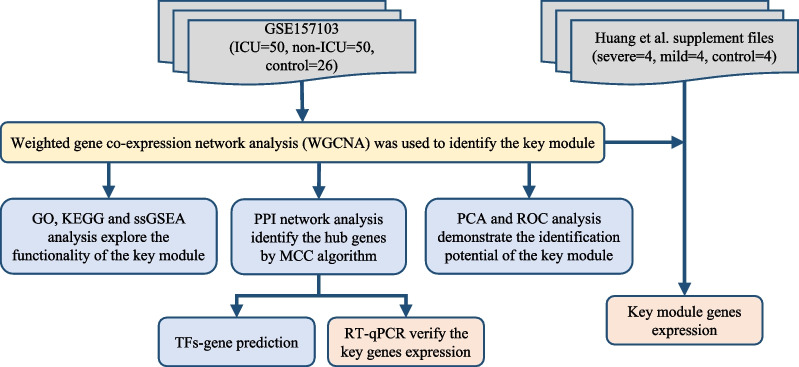
Schematic flow chart demonstrating the process of the analysis

**Fig. 2:**
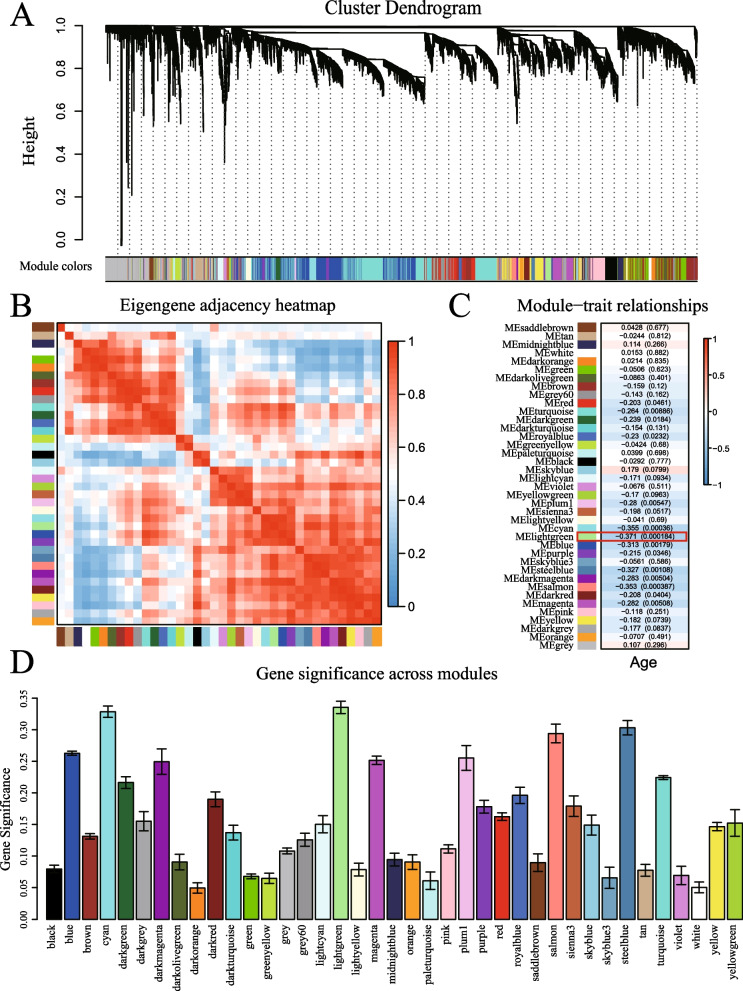
Results of Weight Gene Co-expression Network Analysis (WGCNA) in 100 COVID-19 patients of different ages. **A** Clustering dendrogram of 10,985 co-expression genes (TPM > 1) based on topological overlap. **B** Eigengene adjacency heatmap of different gene co-expression modules. **C** Module-age correlative analysis. Each row corresponds to a module eigengene and each column corresponds to different ages. Heatmap block with *p*-values and correlation coefficients. The red box in the figure shows the module with the highest correlation coefficient in the age range. **D** The barplot shows the absolute value of the correlation coefficient of each module for different ages

We developed a flowchart to systematically analyze the age-related genes in COVID-19 (Fig. 1). First, 10,985 genes (TPM > 1) in the 100 samples of COVID-19 patients were used to set up the co-expression network. Three outliers which found by samples clustering (Additional file 1: Fig. S1A) were eliminated in order to make the subsequent analysis more reliable. To construct the network, a soft-threshold of 14 was used to obtain the approximate scale-free topology (Additional file 1: Fig. S1B, C). Genes across the 97 samples were hierarchically clustered based on topological overlap (Fig. 2A). 39 modules were identified in which genes are co-expressed and random colours were allotted to distinguish different modules. To examine the relation of these modules, we built an eigengene adjacency matrix by calculating the correlation of the eigengenes matrix. Correlation between modules were represented by heatmap (Fig. 2B), and it indicated relative independence among these modules.

For the purpose of determining if any of the identified co-expression modules were associated with age, the correlation coefficient were calculated between each module and aging status by correlating the eigengene values of each module with the age trait (Fig. 2C). We found more modules were negatively correlated with age phenotype, and only a few were positively correlated with this trait. Barplot was used to further represent gene significance across modules (Fig. 2D). The results demonstrated that age was most significantly correlated with the lightgreen module, which composed of 108 genes, suggested those 108 genes were mostly associated with the age-related phenotypes in COVID-19 patients. Therefore, this module can be adopted to represent the ageing stage of COVID-19 patients. For those, we selected lightgreen module for further investigation and will use the key module to refer to them.

### Key module was shown to correlate with T cell function

**Fig. 3:**
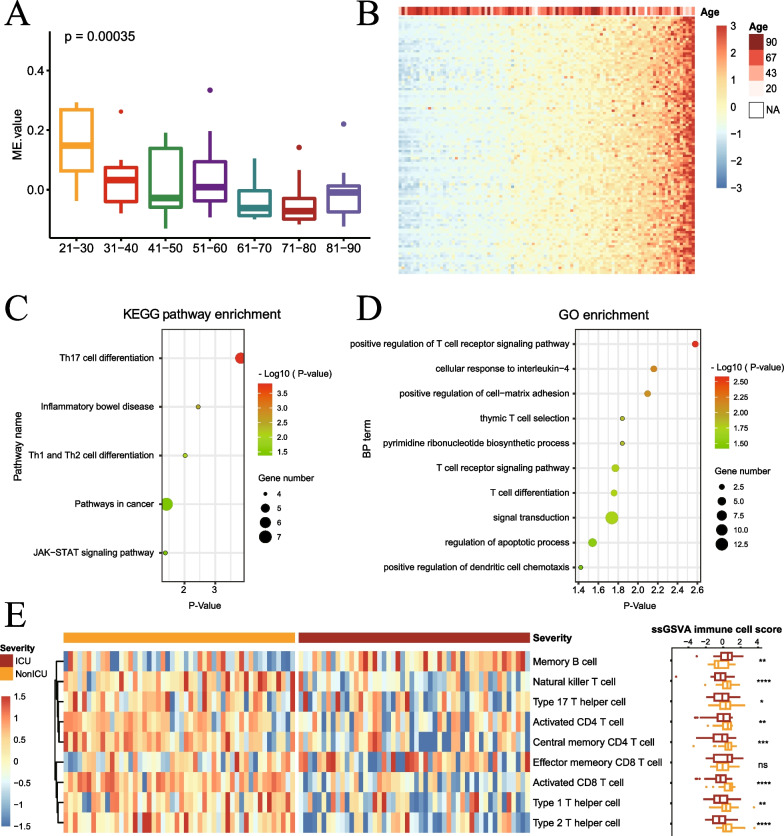
Lightgreen module gene features. **A** Variation analysis of the lightgreen module eigengene values in different age groups (*p*-value = 0.00035). **B** Gene expression heatmap of 93 genes in the lightgreen module in COVID-19 patients with different ages. **C** KEGG pathway enrichment analysis of genes in the lightgreen module. **D** GO enrichment analysis of genes in the lightgreen module. **E** Differential enrichment scores of 9 immune cell signatures among ICU group and non-ICU group

To further investigate the relationship between key module’s gene expression and distribution in COVID-19 patients, patients were divided into 7 age groups every 10 years [37]. By performing the eigengene variation analysis (Fig. 3A), we found module eigengenes (ME) values were generally higher in younger patients than in older patients (*p* = 0.00035). In general, the ME value gradually decreased with the increase of age. The gene expression was further analysed and results revealed that higher expression of key module genes in young adults, while lower expression in aged patients (Fig. 3B).

In order to further explore the function of the key module. Enrichment analysis were performed by DAVID platform [22]. KEGG pathway enrichment analysis showed that T cell-related categories, including Th17 cell differentiation and Th1 and Th2 cell differentiation, were enriched in these key module genes (Fig. 3C). We also performed GO enrichment analysis (Fig. 3D). The key module genes were also significantly enriched in T cell related pathways, such as positive regulation of T cell receptor signaling pathway, thymic T cell selection, T cell receptor signaling pathway and T cell differentiation. It suggested that T cell-related genes expressed variant in different age groups of COVID-19 patients. We further characterized the immune cell components in 100 COVID-19 patients by scoring key module genes using ssGSEA (Fig. 3E). Interestingly, Key module genes were scored mainly in T cells subtypes, including natural killer T cell, type 17 T helper cell, activated CD4 + T cell, central memory CD4 + T cell, effector memory CD8 + T cell, activated CD8 + T cell, type 1 T helper cell and type 2 T helper cell. Nevertheless, the immune cell composition was significantly different among 50 ICU-patients and 50 non-ICU-patients. Except effector memory CD8 + T cell which was not significant, the composition of other subtypes of T cells was higher in non-ICU patients than in ICU patients while memory B cell was the opposite. Thus, T cell depletion characterizes severe COVID-19 disease.

### Key module genes can distinguish COVID-19 patients between different status

**Fig. 4:**
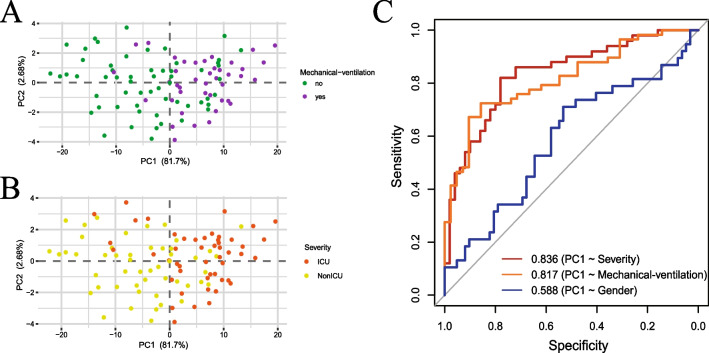
Performance of lightgreen module analysis. **A**, **B** Principal component analysis (PCA) of lightgreen module. Each dot represents one sample. Green: non-mechanical-ventilation. Purple: mechanical-ventilation. Orange: ICU. Yellow: non-ICU. **C** Receiver operating curve (ROC) plot of the performance based on accuracy using PC1 of lightgreen module genes for severity, mechanical ventilation and gender

To further determine the power of key module in COVID-19 patients, principal component analysis (PCA) was proceeded to construct the key module signatures. We selected the top two significant components with the higher contribution degree, which explained 81.7% and 2.68% of the key module variation, to identify COVID-19 patients’ different status. Surprisingly, the first principal component (PC1) mainly separated mechanical-ventilation patients from non-mechanical-ventilation patients (Fig. 4A), and it also effectively separated ICU patients from non-ICU patients (Fig. 4B). We also analysis the gender distribution in two dimensions by the top two significant components (Additional file 2: Fig. S2A), just like we thought, there was little discrimination between gender. Taken together, measures mentioned above confirmed the importance of the key module in COVID-19 status.

Then, principal component 1 was selected to act as a signature score predicting COVID-19 status on the basis of its highest contribution degree. To further explore the diagnostic potential of the key module, we perform receiver operating characteristic (ROC) curve analysis. The area under the curve (AUC) for discriminating whether required to ICU was 0.836, and for discriminating whether used mechanical ventilation was 0.817 (Fig. 4C). For contrast, we demonstrated the diagnostic power of PC1 for gender, and the AUC was only 0.588. This suggests that the PC1 has a strong diagnostic capacity for patients’ severity and mechanical-ventilation and there is little discrimination for gender. To further demonstrate the potential capabilities of age-related key module, rather than the role of age itself. We also performed ROC analysis by replacing PC1 with age, and analyzed the diagnostic power of age for severity, mechanical-ventilation and gender (Additional file 2: Fig. S2B). The AUC were 0.548, 0.475 and 0.494, suggesting age-related key module has high diagnostic potential for severe COVID-19, while age cannot predict the severity of patients. Because of the correlation between the key module and T cell-related functions, we assumed that alteration of the key T cell-related gene expression may be a reason for elderly COVID-19 patients progressed to severe disease with an unfavourable outcome.

### Ten genes were identified as hub genes and three genes were considered as key TF-genes

**Fig. 5:**
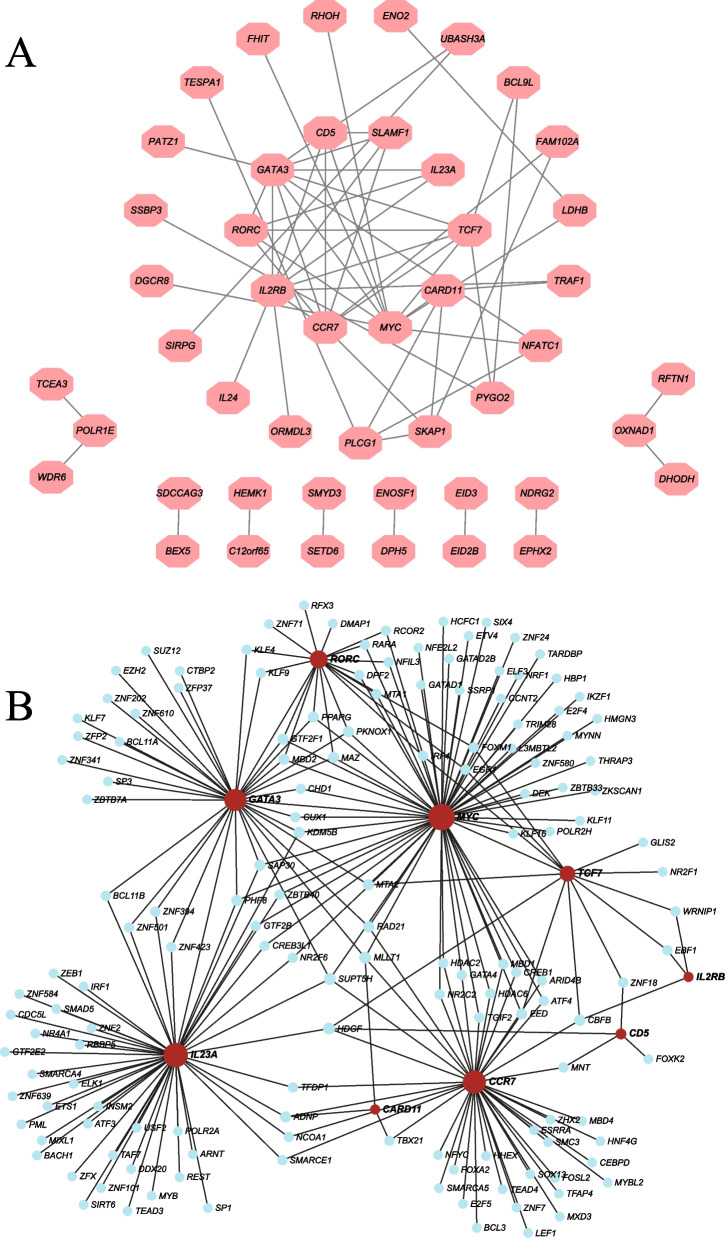
Network analysis. **A** PPI network diagram of lightgreen module genes. The network diagram contains 47 nodes and 60 edges. The first 10 genes are selected as *GATA3*, *CCR7*, *IL2RB*, *CD5*, *SLAMF1*, *TCF7*, *MYC*, *RORC*, *IL23A* and *CARD11*. **B** Transcriptional factors-genes network diagram. The red circle represents the hub genes of PPI, and the blue circle represents the transcription factor

The PPI network was constructed with 108 key module genes using the STRING database [29]. The network diagram contains 47 nodes and 60 edges (Fig. 5A). By MCC algorithm [38], the network interaction was presented among these genes and identified 10 hub genes (the core components of the module that were representative of the module’s function), including *GATA3*, *CCR7*, *IL2RB*, *CD5*, *SLAMF1*, *TCF7*, *MYC*, *RORC*, *IL23A* and *CARD11*, based on a higher degree of connectivity in the key module.

TFs–genes interactions showed the interaction between hub genes selected above by using NetworkAnalyst. The TFs–genes interaction network consisted of 157 nodes and 448 edges (Fig. 5B). Among them, *MYC* was regulated by 62 TF genes, *IL23A* was regulated by 48 genes, *CCR7* was regulated by 41 genes, *GATA3* was regulated by 35 genes and *RORC* was regulated by 19 genes. Among these TFs-genes, *HDGF*, *SUPT5H* and *MLLT1* with 4 edges was considered as key TF-genes which was highly related with regulation of T cell differentiation in elderly COVID-19 patients.

### Validation of the key genes revealed significant differences in the expression of genes related to T cell differentiation in different age groups

**Fig. 6:**
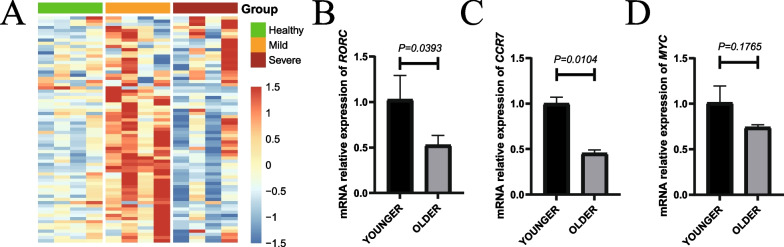
Independent data sets and RT-qPCR experiments. **A** Heatmap represent the key module genes in independent data sets. **B**, **C**, **D** Validation of the expression level of three key genes using RT-qPCR: **B** *RORC*, **C** *CCR7* and **D** *MYC.* unpaired Student’s t-test

To validate our results, we analysed RNA-seq data from other independent COVID-19 datasets in vivo and observed the same results (Fig. 6A). The expression of key module genes was higher in the younger group. To further validate the aforementioned bioinformatics analysis, we had consulted additional literature and found more evidence of the association of *RORC*, *CCR7*, and *MYC* with COVID-19 and T cells before we performed laboratory experiments [39–47]. The mRNA expression levels of these genes were obtained by the RT-qPCR experiment in key genes which was highly ranked in relation to transcription factors and associated with important phenotype (Fig. 6B, C, D). In different age groups, the mRNA relative expression of *RORC* and *CCR7* gene were significantly higher in younger group compared with older group (*RORC*: *p* = 0.0393, *CCR7*: *p* = 0.0104). *MYC* had the same trend although there was no statistical difference (*MYC*: *p* = 0.1765).

## Discussion

Due to the pandemic of COVID-19, millions of people are severely affected. More and more people passed away, especially among the elderly. Even though several drug candidates and vaccines have been researched and applied to treat the patients, there is still no reliable treatments for elderly. Inspecting gene co-expression patterns is proven to be an effective method to analyse and uncover complicated genetic network. In our study, gene co-expression analysis was performed on RNA-sequencing (RNA-seq) data set, which contained gene expression data from 100 COVID-19 patients. We identified a key module, consisting of 108 genes, which is most associated with age phenotypes. The results of the enrichment analysis suggested that the key module and the underlying pathological processes of the disease were associated with T cell-related pathways.

Carolyn et al. focused on the different SARS-CoV-2 specific T cell responses between in children and adults and found low T cell responses in children [11]. We focused on young and old patients and hypothesised that T cell differentiation played a key role in different outcomes of different age groups of COVID-19 patients. Our results may complement those of Carolyn et al. T cell differentiation is not only different in children and adult, but also in different age groups of adult patients with COVID-19. An age-dependent stratification of severe COVID-19 patients was also reported in previous study [48]. Other researches have shown that severe COVID-19 patients have a significant age-associated increase of autoantibody levels against 16 targets (e.g., amyloid β peptide, β catenin, cardiolipin, claudin, enteric nerve, fibulin, insulin receptor α, and platelet glycoprotein). These findings have provided key new insights into the reason why the prognosis for older patients is worse compared with younger ones [48].

T cells play a vital role in viral clearance, but the underlying pathophysiology of T cells in COVID-19 is extremely complex [49]. It has been reported that CD8 + cytotoxic T cells secrete a range of molecules such as perforin, granzymes, and IFN-γ to remove viruses from the host [50]. At the same time, CD4 + helper T cells (Th cell) assist cytotoxic T cells and B cells, enhancing their ability to clear pathogens [51]. However, continuous viral stimulation may induce T cell failure, resulting in loss of cytokine production and reduced function [52, 53]. Earlier studies have been unclear regarding the numbers and function of T cells in COVID-19 patients, albeit with suggestions of depressed lymphocyte counts [54, 55]. Diao et al. recently reported that the novel coronavirus could trigger the release of cytokines which in turn drive T cells depletion and exhaustion, instead of attacking T cells directly. Beside, they also found the number of T cells was negatively correlated with case severity [56]. In our study, we identified a key module, consisting of 108 genes through 100 COVID-19 patients. And we found immunocyte enrichment scores of key module genes were higher in the mild group than in the severe group. It is consistent with the results of Diao et al. [56], and also positive for the reliability of the key module.

Our findings also highlight the strong discrimination ability of the key module for mild and severe cases of COVID-19. As we all know, ICU and mechanical ventilation are important indicators in severe respiratory disease. And there was no direct correlation between gender and age-related module. At the same time, we demonstrate that age is not an independent determinant of disease progression. At present, the global rate of severe cases of COVID-19 is gradually decreasing, which may be due to the fact that doctors have more experience in the diagnosis and treatment of COVID-19 as time goes on. The mutation of the virus may also lead to the gradual weakening of virulence. Of course, all of this has yet to be further confirmed. Nevertheless, the progress of elderly patients to severe disease still poses a great burden to society. The hub genes and TFs we identified may help further reduce the rate of severe progression in older patients.

Our approach is markedly different from previous bioinformatics reports in COVID-19 studies [57–60], which relied on identifying genes by differential analysis. However, in order to provide insights into systems biology, we implemented gene co-expression module analysis to provide key gene modules instead of looking for differential gene expression (DEGs). We clustered age-related genes and detected hub genes according to the highly connected nodes in each module network. Lightgreen module was identified as the key module with the highest level of significant association. And Ten genes in the key module were further identified as hub genes, including *GATA3*, *CCR7*, *IL2RB*, *CD5*, *SLAMF1*, *TCF7*, *MYC*, *RORC*, *IL23A* and *CARD11*. Most of these 10 hub genes have been reported to be significantly associated with T cell differentiation. For example, *CCR7* activated mutations in T cell receptor-NF-κB signaling, T cell trafficking and other T cell-related pathways [61]. Expression of the Th17 transcription factor *RORC* was high in rheumatoid arthritis [62]. *CCR7*, *RORC* and *MYC* were not only associated with T cell differentiation, but also considered to be transcription factors that regulate many downstream immune-related or T cell-related genes [62–65]. These 10 hub genes may have the most important effect on outcomes in different age COVID-19 patients through regulating downstream key module genes. However, how those hub genes control the key modules needs further investigation and requires more examinations.

The TFs-genes network showed that *HDGF*, *SUPT5H* and *MLLT1*, with the most edges, played important roles in age-related processes of COVID-19. To our surprise, these key TFs were associated with T cells. For instance, *HDGF* can induce the immune suppressor functions on CD8( +) T cell activities [66]. *SUPT5H* was positively correlated with activated memory CD4 + T cells [67]. *MLLT1* is down-regulated in NK cells expressing T cell immunoglobulin [68]. These TFs had been studied in kinds of diseases, particularly in cancer, but the role of these genes in advanced age COVID-19 patients need further study.

Zhao et al. published a study by single-cell omics which revealed T cell immune response in severe COVID-19 patients [69]. They suggested tissue-resident memory-like Th17 cells were associated with disease severity and lung damage. As mentioned above, *RORC* gene is related to Th17 cells. Meanwhile, Hassaniazad et al. showed that *RORC* is related to the recovery of acute inflammatory response of COVID-19 [39]. Therefore, *RORC* was identified as an experiment-candidate gene. We also found *CCR7* shares edges with all the key TFs (*HDGF*, *SUPT5H* and *MLLT1*) identified in this study. It was reported that the specific marker *CCR7* of immune cell subsets in critically ill patients was significantly lower than that in healthy case [44] and specific T cells trend to differentiate to *CCR7*-*CD45RA* + effectors after exposure to SARS-CoV-2 antigen [45]. In addition, *MYC* has the largest number of TFs connected in our study. T cell proliferation is closely related to *MYC* gene expression [70, 71], and coronavirus infection can affect *MYC* gene expression [47]. Therefore, we performed RT-qPCR of *RORC*, *CCR7* and *MYC*. It showed that the expression of *RORC* and *CCR7* in PBMC in younger group (20–30 years old) was significantly higher than older group (40–50 years old). The expression of *MYC* in younger group was higher than that older group, the difference was not statistically significant. We considered the individual differences in *MYC* may be due to more transcription factors and more complex regulatory networks.

Our analysis focused on PBMC gene expression analysis to obtain further insights regarding the potential utilization of the identified hub genes in diagnostic development for COVID-19. Our analysis shows that the key module has excellent diagnostic capabilities. This may have potential in the future diagnosis and treatment of mild and severe cases of COVID-19. However, several limitations of the study should be noted as expanding the sample size will be more helpful to our conclusion. In addition, it has been reported that the most striking phenotypic differences between young and old humans in immune parameters is the distribution of T cell differentiation phenotypes [72]. This also provides evidence for our conclusion. Changes in T cell differentiation-related genes play a key role in the outcomes of COVID-19 patients in different age groups. Our conclusion will be useful for the development of therapeutic strategies in elderly COVID-19 patients and give us a hint that we should pay more attention to research about T cell differentiation status in aged COVID-19 patients.

## Conclusions

In conclusion, we identified 10 age-related genes associated with the COVID-19 patients’ status, and constructed a TFs-genes interactions network of the hub genes. And we discovered age-related genes were associated with T-cell function. These biomarkers can properly predict the status of COVID-19 patients. Further studies should be performed to explore the precise role of these genes in COVID-19.

## Supplementary Information

**Additional file 1: Fig. S1.** Co-expression construction. **A** Sample clustering dendrogram. The outliers are GSMA4753070, GSMA4753095 and GSMA4753087. **B** The relationship between soft-threshold (power) and scale-free topology. **C** The relationship between soft threshold (power) and mean connectivity

**Additional file 2: Fig. S2.** Performance of the key module. **A** Principal component analysis (PCA) of lightgreen module genes and gender. Each dot represents one sample. Red: female. Blue: male. **B** Receiver operating curve (ROC) plot of the performance based on accuracy using age for severity, mechanical ventilation and gender

## Data Availability

Whole transcriptome sequencing data and clinical data of the 100 COVID-19 cohort mentioned above were download from the SRA database SRP279280 (https://www.ncbi.nlm.nih.gov/sra/) and supplement material of published literature.

## Abbreviations

AUC: Area under the curve
BH: Benjamini–Hochberg
CORUM: The comprehensive resource such as mammalian protein complexes
COVID-19: Corona virus disease 2019
DEGs: Differential gene expression
ECDF: Empirical cumulative distribution function
GO: Gene ontology
ICU: Intensive care unit
MCC: Maximal clique centrality
ME: Module eigengenes
PBMC: Peripheral blood mononuclear cell
PC: Principal component
PCA: Principal component analysis
PPI: Protein–protein interaction
ROC: Receiver operating characteristic
SARS-CoV-2: Severe acute respiratory syndrome coronavirus 2
SRA: Sequence read archive
ssGSEA: Single-sample GSEA
TFs-gene: Transcription factors-gene
Th cell: Helper T cells
TOM: Topological overlap matrix
TPM: Transcript per million
WGCNA: Weighted gene co-expression network analysis
