# Supplementary figures and images for: Weighted gene co-expression network analysis revealed T cell differentiation associated with the age-related phenotypes in COVID-19 patients
Source: BMC Med Genomics. 2023 Mar 25;16:59. doi: 10.1186/s12920-023-01490-2 (PMC10039774; doi:10.1186/s12920-023-01490-2)

A

## Sample clustering to detect outliers

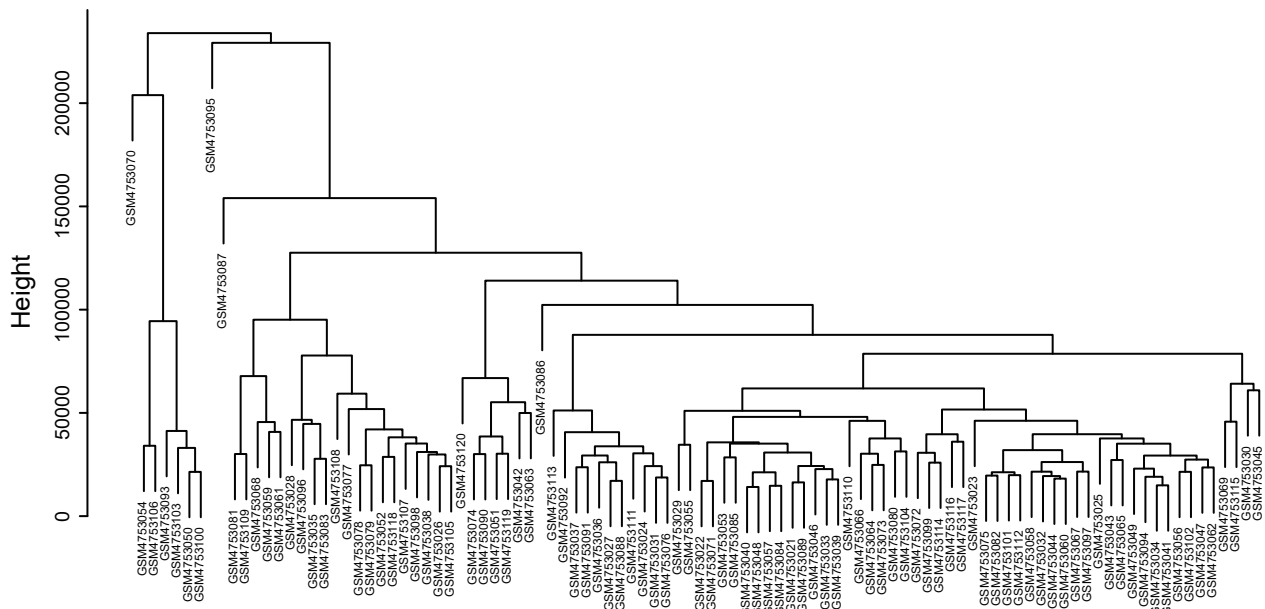

B

## Scale independence

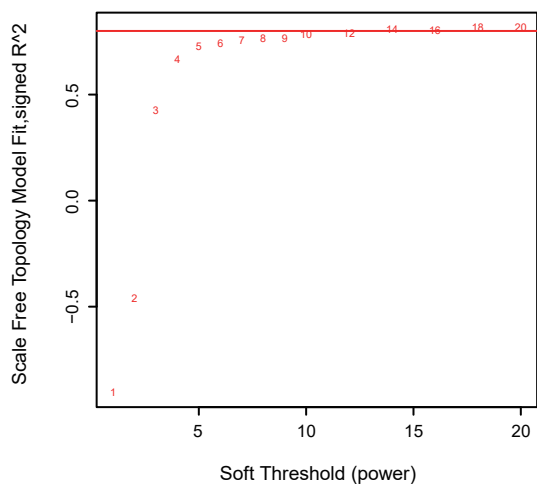

C

## Mean connectivity

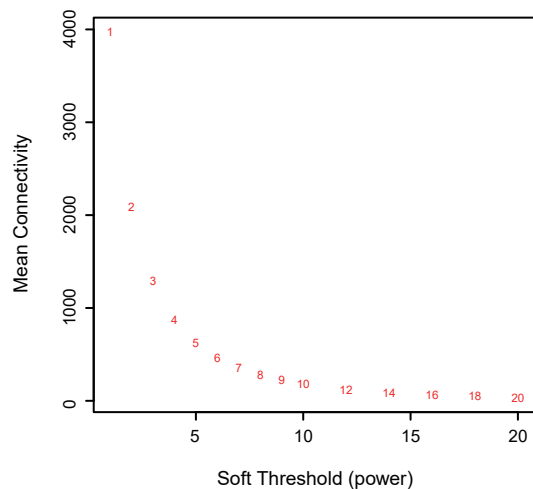

Supplement: Supplementary file 1 — Additional file 1: Fig. S1. Co-expression construction. A Sample clustering dendrogram. The outliers are GSMA4753070, GSMA4753095 and GSMA4753087. B The relationship between soft-threshold (power) and scale-free topology. C The relationship between soft threshold (power) and mean connectivity [file 12920_2023_1490_MOESM1_ESM.pdf]

A

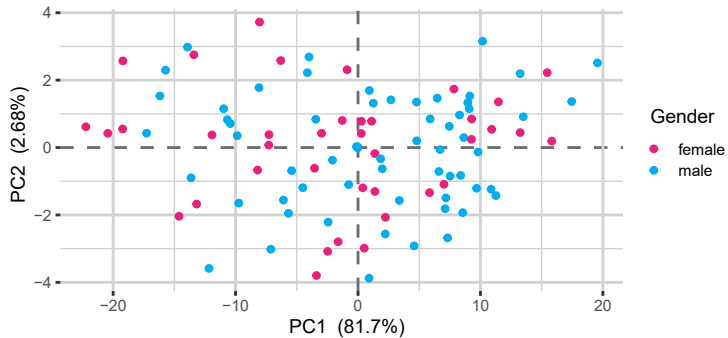

B

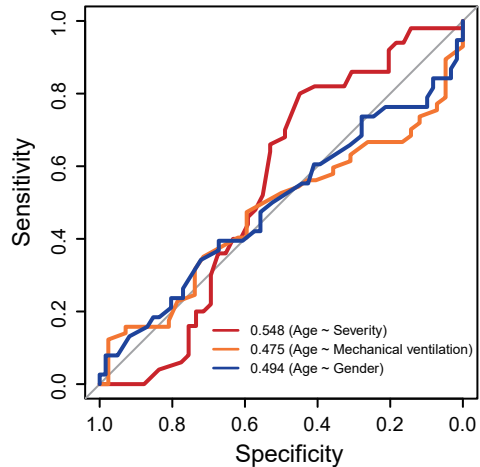

Supplement: Supplementary file 2 — Additional file 2: Fig. S2. Performance of the key module. A Principal component analysis (PCA) of lightgreen module genes and gender. Each dot represents one sample. Red: female. Blue: male. B Receiver operating curve (ROC) plot of the performance based on accuracy using age for severity, mechanical ventilation and gender [file 12920_2023_1490_MOESM2_ESM.pdf]
